# Rights-Based Training Enhancing Engagement of Health Providers With Communities, Cape Metropole, South Africa

**DOI:** 10.3389/fsoc.2019.00035

**Published:** 2019-04-30

**Authors:** Gimenne Zwama, Maria Clasina Stuttaford, Hanne Jensen Haricharan, Leslie London

**Affiliations:** ^1^Health and Human Rights Programme, School of Public Health and Family Medicine, University of Cape Town, Cape Town, South Africa; ^2^Institute for Global Health and Development, School of Health Sciences, Queen Margaret University, Edinburgh, United Kingdom; ^3^Health, Social Care and Education, Kingston and St George's University of London, London, United Kingdom

**Keywords:** training, health providers, community participation, health committees, governance, PHC, rights, South Africa

## Abstract

Community participation, the central principle of the primary health care approach, is widely accepted in the governance of health systems. Health Committees (HCs) are community-based structures that can enable communities to participate in the governance of primary health care. Previous research done in the Cape Town Metropole, South Africa, reports that HCs' potential can, however, be limited by a lack of local health providers' (HPs) understanding of HC roles and functions as well as lack of engagement with HCs. This study was the first to evaluate HPs' responsiveness towards HCs following participation in an interactive rights-based training. Thirty-four HPs, from all Cape Metropole health sub-districts, participated in this qualitative training evaluation. Two training groups were observed and participants completed pre- and post-training questionnaires. Semi-structured interviews were held with 10 participants 3–4 months after training. Following training, HPs understood HCs to play an important role in the communication between the local community and HPs. HPs also perceived HCs as able to assist with and improve the quality and accessibility of PHC, as well as the answerability of services to local community needs. HPs expressed intentions to actively engage with the facility's HC and stressed the importance of setting clear roles and responsibilities for all HC members. This training evaluation reveals HPs' willingness to engage with HCs and their desire for skills to achieve this. Moreover, it confirms that HPs are crucial players for the effective functioning of HCs. This evaluation indicates that HPs' increased responsiveness to HCs following training can contribute to tackling the disconnect between service delivery and community needs. Therefore, the training of HPs on HCs potentially promotes the development of needs-responsive PHC and a people-centred health system. The training requires ongoing evaluation as it is extended to other contexts.

## Introduction

With global attention for people-centred health systems gathering momentum and the World Health Organisation's (WHO) publication of its global strategy on people-centred services in 2015, it is widely emphasised that not only service users, but also communities, should play an active and informed role in the maintenance, restoration, and promotion of their own health (Hunt and Backman, 2007; WHO, 2015). The Alma-Ata Declaration stresses that people have the right and duty to participate in the planning, organisation, operation and control of primary health care (PHC), and builds on the right to health, adopting the WHO's definition of health as “the state of complete, physical, mental, and social well-being, and not merely the absence of disease or infirmity” (WHO, 1946, 1978). Community participation in the governance of service delivery can promote people-centeredness and needs-responsiveness of a health system (WHO, 1978, 2015). These are characteristics of a health system in which everyone contributes and benefits and where health care services respond to people's needs and expectations in a holistic manner, rather than focusing solely on disease and the diseased (WHO, 2007, 2015).

For people's participation to be effective and meaningful, communities' active and informed involvement is required in the evaluation of strategies, decision-making, prioritisation, and implementation of the right to health (Potts, 2008). In a systematic review of evidence on Health Committee (HC) effectiveness in low- and middle-income countries (LMICs), HCs have commonly been found to provide a bottom-up platform for community representatives to participate in health care decision-making, monitoring and oversight (McCoy et al., 2012). In Kenya, HCs are official structures with defined roles to close gaps in service delivery and to hold health facilities accountable for the quality and accessibility of the services offered (Goodman et al., 2011). By these means, HCs can facilitate the community's collective ownership of PHC services (Haricharan, 2012) as well as promote the realisation of the right to health (Glattstein-young, 2010; Chikonde, 2017). As HCs serve as community-based governance structures in the delivery of primary health services, they are inherently interdependent on the dynamics of the health system's social, economic, and political contexts (United Nations Committee on Economic Social Cultural Rights UNCESCR, 2000; Gilson and WHO, 2012). This requires us to investigate such contextual factors and cross-cutting issues that can challenge HC functioning. In their reviews, George et al. (2015a) and McCoy et al. (2012) stress the importance of contextual influences in understanding HCs' role and contribution to health systems strengthening.

A cross-case comparative study of 11 HCs in West and Central Africa found that HCs' individualised and non-systematic character can leave marginalised groups excluded (Lodenstein et al., 2017b). Even when HC powers and roles in accountability are partially defined on a national level, full specifications of these powers and tools to execute them are needed (Lodenstein et al., 2017b). The South African National Health Act (NHA) states that a HC must be composed of community members, a local government councillor and a health facility manager (The Republic of South Africa, 2004). The Department of Health, however, delegates the definition of HC role and mandate to provincial policy legislation and action. All provinces currently have legislation, draft legislation, or guidelines, which differ substantially in the nature and extent to which HC roles and responsibilities are described (Haricharan, 2013). Accordingly, it was found that Provincial Departments of Health can fall short in their guidance, direction and training of HCs, thereby negatively impacting HCs' effective functioning (Padarath and Friedman, 2008; Meier et al., 2012). This lack of specification on health committees' roles and functions, as well as HCs' lack of power and legal mandate, can limit their uniform functioning and effective integration within the health system (Padarath and Friedman, 2008; Haricharan, 2012; Boulle, 2013).

In addition, there appears to be a general lack of clarity and guidelines on HC member election procedures and the make-up of the electorate. In South Africa, the Eastern Cape is the only Province that fully specifies the election of HC members through a representative democratic process in their final draft policy on HCs (Eastern Cape Department of Health, 2009). However, this policy defines HC community members as representatives from organised community initiatives, thereby possibly compromising the HC member's representativeness of community members. A HC training manual developed for the South African context, encourages and defines the procedure of HC member election by the rules of the Constitution (The Learning Network for Health and Human Rights, 2014).

In comparison to other South African Provinces, the Western Cape had lagged behind in passing HC legislation (Haricharan, 2013). Policy developments lost momentum after the Head of Health for the City of Cape Town, a HC champion, passed away in 2008. The loss of this champion was a critical milestone alongside the broader complexities of the South African health (committee) policy context over time. The long-awaited draft Bill on Health Facility Boards and Committees was published in 2015 (Province of Western Cape, 2016). This Bill became an Act in 2016 and is yet to be implemented. Even though the Act recognises HCs as a community platform, it significantly reduces the scope of their role in decision-making, strategizing, prioritising, and implementing health services according to local needs. Secondly, it allows for the provincial Minister of Health to elect committee members, which can pose a threat to the democratic value of HCs.

The national South African Department of Health committed to the “re-engineering” of PHC in 2010 with the purpose to improve service quality and integration (Barron et al., 2010). This “re-engineering” was to be established by holding the management of the district health system responsible for meeting “key ministerial priorities,” implying a top-down, non-democratic process. In June 2018, the cabinet approved the National Health Insurance Bill with the goal to provide all South Africans “access to quality and affordable health care services based on their health needs irrespective of their socio-economic status” (The Republic of South Africa, 2018). Strikingly, considering its purpose, the National Health Insurance Bill does not acknowledge the potential of HCs as a platform for community participation, nor community participation as a continuous mechanism to identify these health needs.

Discrepancies between policy and practice can compromise HCs main role as an intermediary between the community and health services (McCoy et al., 2012; George et al., 2015a). As evaluations across East and Southern Africa and from Brazil suggest, such discrepancies can influence nurses and facility managers' capacity to work with HCs and require their education on HCs' roles and functions (Zambon and Ogata, 2011; Loewenson et al., 2014; George et al., 2015a). George et al. (2015b) reported that poor interpersonal skills, lack of training, perceived lack of skill and lack of trust of providers were challenges for community participatory platforms to improve the quality of services.

For HCs to effectively integrate into and contribute to a people-centred health system, HPs play an important role in creating a responsive environment in which communities meaningfully participate. McCoy et al. (2012) and George et al. (2016) illustrate that there has been a predominant focus on the capacity building of communities. While there are a few studies highlighting both sides of the coin (Mosquera et al., 2001; Sohani, 2005), there is an evident gap in research on the impact of HP training on HCs' effective and meaningful participation. Research in South Africa has shown that while some facility managers are aware of HC roles and functions and attend their meetings, others are completely unaware (Padarath and Friedman, 2008; Haricharan, 2012; Boulle, 2013). As a result, HPs can be reluctant to involve communities and find it challenging to be held accountable by the community, or perceive HCs solely as an extension of service delivery (Padarath and Friedman, 2008; Glattstein-young, 2010; Haricharan, 2012; Boulle, 2013).

The Learning Network for Health and Human Rights, a collaboration of civil society organisations and two universities in the Western Cape, aims to promote the right to health through community participation. In the Cape Metropole, HC-HP engagement was found to be challenged by untrained community members, power imbalances, lack of mutual trust as well as HPs' lack of understanding of the relationship between the right to health and participation (Haricharan, 2012). As part of their activities to fulfil this purpose, the Learning Network trained nearly 300 HC community members across the Cape Metropole (Haricharan, 2017). An evaluation of this HC training indicated that although HC training can improve levels of participation, this is influenced by HP authority as well as power imbalances between HC members (Chikonde, 2017). As HPs had not been trained, a HP training manual was informed by HC members, developed and piloted with the aim to (re-)establish and strengthen HPs' working relationships with HCs.

Building on everyone's right to health and participation, this study evaluated a rights-based, interactive training of HPs on HCs. This paper reports the extent and nature to which HPs' immediate and short-term responsiveness changed as a result of the training. It sheds light on contextual factors that can be of influence on HPs' ability to implement their responsiveness. Findings are discussed and their potential contribution to the promotion of community participation in the strengthening of people-centred health systems is described.

## Methods

### Socio-Economic Profile

In 2018, the Cape Metropole population was estimated to reach 4.06 million (Western Cape Government, 2018). The Cape Metropole is the least unequal Metropoles of South Africa with a Gini coefficient of 0.58. For the next 5 years, the City of Cape Town estimates that the aged population over 65 will increase at 3.4 percentage per year. This, while the child cohort (age 0–14 years) will grow by 1.2% and the working age population by 0.8% per year. With an additional unemployment rate of 11.9 percentage, this can be expected to pose a greater burden on social systems and basic service delivery. In line with the disease burden, PHC facilities currently offer testing and treatment of HIV, STIs, tuberculosis, diabetes, and hypertension as well as immunisation and child health services. To varying extents, facilities also offer maternal and mental health services.

### Training Purpose and Approach

The training manual, compiled for the purposes of the rights-based training evaluated in this research, titled “*Community Engagement for Quality Care”* consists of two main chapters called “Relationship Building” and “Health Committees and Governance” (Marshall and Mayers, 2015). In line with the NHA (The Republic of South Africa, 2004), this training aimed to promote health services that are responsive to community participation. It facilitated reflection on the dual obligation and responsibilities of health care providers towards the State by referring to the national vision to achieve a society committed to democratic values, social justice, and fundamental human rights as well as the rights of the patient as set out in the Batho Pele or “People First” service principles (The Republic of South Africa, 1996, 1997).

The training adopted an experiential learning approach (Kolb, 1984), whereby the facilitators guided participants through a reflective learning process that shed light on previous and current practices of engaging with, and involving the community. The importance of mutual understanding, collaboration, and respect was illustrated through rights-based case discussions, role plays and reflections on the values of compassion, and professionalism. In these ways, tailored directions could be given and environments promotive of the right to health and participation could be illustrated.

### Training Implementation and Participant Recruitment

Thirty-four health care providers from all City of Cape Town health sub-districts (*n* = 8) were recruited for the training, contributing to the diversity of the study sample (see Table 1). These included (senior) professional nurses and clinic managers working in City of Cape Town clinics, as well as environmental health practitioners, health promotion officers, and programme officers working at sub-district level (see Table 2). Six sub-districts had at least four participants attending the training, of which two sub-districts had eight representatives each. However, one of the remaining sub-districts was represented by two participants both positioned at sub-district level and the other by two environmental health practitioners. Each of the two training groups had one male attendee, both environmental health practitioners.

**Table 1:** Number of participants and sub-districts.

	**Training observations, in % (*n*)**	**Pre-questionnaire responses, in % (*n*)**	**Post-questionnaire responses, in % (*n*)**	**Interviews, in % (*n*)**	**Sub-districts**
Training group 1	58.8 (20)	85.0 (17)	85.0 (17)	25.0 (5)	A, B, C, D
Training group 2	41.2 (14)	100 (14)	85.7(12)	35.7 (5)	D, E, F, G, H
Total	100 (34)	91.2 (31)	85.3 (29)	29.4 (10)	100 (8)

**Table 2:** Number of participants by professional position and sub-district of origin.

**Position**		**Questionnaire responses, in % (*n*) *sub-district***	**Interviews, in % (*n*) *sub-district***
*Clinic level*	Clinic manager	48.4 (15) *A, C, D, E, F, G*	40.0 (4) *A, C, F, G*
	Senior professional nurse	16.1 (5) *A, C, E, F*	20.0 (2) *C, E*
	Professional nurse	12.9 (4)^*^ *A, D, E*	10.0 (1) *D*
*Sub-district level*	Environmental health practitioner	9.7 (3) *A, H*	10.0 (1) *H*
	Health Promotion Officer	6.5 (2) *B, D*	20.0 (2) *B, D*
	Programme officer	6.5 (2) *B, D*	–

* *Two missing post-questionnaires, both professional nurses from district E*.

Initially, the training was intended to consist of 2 consecutive days. However, several sub-district managers expressed their concern about the burden it could place on the facilities when some of their employees are away from the services for this amount of time. As a result, it was decided to decrease the training to 1 day followed up by another half a day at least a month later. Training sessions were implemented in May and July 2015 for the first (*n* = 20) and second group (*n* = 14) of participants, respectively. These 1-day training sessions explored the influences on and key elements for relationship building with communities as well as HC composition, roles, and functions. Preliminary analysis of questionnaires and observations was used to feed into the agenda of the follow-up session. Accordingly, this session intended to further develop HP skills for relationship building with HCs, in particular on how to establish a common vision, host a HC meeting, and manage conflict. In addition, it would provide an opportunity to further explore and discuss power imbalances and other practical issues raised by participants.

Follow-up sessions were scheduled for ~6 weeks after the first training sessions. Only two out of 10 enrolled participants attended the follow up training session, both holding a position at sub-district level. The first groups' participants cancelled or did not attend the follow up for various reasons such as conflicting meetings or courses, deadlines for end of financial year reports, and staff shortages due to seasonal illness. A rescheduled, combined follow up session was cancelled due to a low confirmed number of attending participants. The majority of participants expressed interest and enthusiasm for the follow-up session and indicated to be disappointed that it did not take place.

With South Africa recognising 11 official languages, participants' native languages differed. Most study participants' first language was isiXhosa (*n* = 12), followed by Afrikaans (*n* = 9), English (*n* = 7), and Sesotho (*n* = 2). The central sessions of the training were conducted in English, as this was the commonly spoken language amongst the participants. The first training group's session was facilitated by two expert educators from the University of Cape Town (UCT), one experienced in training, and consulting HCs (also proficient in isiXhosa), the other with experience as an academic teacher and professional nurse (also proficient in Afrikaans). Due to unavailability of the former, the latter facilitator acted as the sole facilitator for the training of the second group.

### Study Design and Data Collection

The design was based on a realist evaluation (Pawson and Tilley, 1997). The evaluation purpose was to explore the possible variations in nature and extent of the immediate and short-term impact of training of HPs on their responsiveness to HCs. In this paper, responsiveness is defined as the collection of understandings, intentions to practices, and practices in support of HC roles and functions. Thereby, we acknowledge that responsiveness is inherently influenced by experiences and contextual factors. The exploration of such factors was facilitated by the training participants' diversity in health care professions, experiences, local contexts, and relationships with HCs. The adoption of a realist approach provided a deeper insight into the facilitating and impeding contextual factors, as well as the dynamic interactions between societal and health systems processes that influence HPs' relationships with HCs and their ability to implement an enhanced responsiveness (Pawson and Tilley, 1997). All data was collected between May and November 2015, and the study adopted a flexible research design making use of pre- and post-training questionnaires, direct observations, semi-structured interviews, and field note journaling.

Field notes were diarised from the moment preparations for training implementation started. Pre- and post-training qualitative questionnaires and a topic guide for semi-structured interviews were developed in consultation and concordance with the facilitators' vision for the training. Due to insufficient time before training implementation, questionnaires were not piloted or cognitively tested. Before distribution, they were evaluated by the second and third author of this paper as well as by the training facilitators. Two attribute-inquiring questions were improved regarding phrasing or ambiguity before distribution to the second group. Before its use, the interview topic guide was adjusted and probes related to questions arisen from analysis of questionnaire responses were added.

Written notes of the training observations were taken. These observations also provided 17.5 h of audio recordings, of which parts were transcribed where relevant to the research questions and where written notes lacked context or clarity. The observations gave insight into the development of changes in HPs' responsiveness and contributed to the triangulation of data.

A total of thirty-one pre-training questionnaires consisting of twenty-one, mostly open-ended, questions were completed by fifteen clinic managers, five senior professional nurses, four professional nurses, three environmental health practitioners, two health promotion officers and two programme officers (Table 2). Four multiple choice questions inquired about the current HC status and relationship at the facility or sub-district level. Furthermore, participants were asked about their understandings of HC roles and benefits, their challenges in engaging and working with HCs as well as the ways in which the health facility can promote HC functioning.

The post-training questionnaires included sixteen questions, of which four open-ended questions to specifically evaluate the training format and content, one multiple-choice question and eleven open-ended questions of which six were similar to the pre-questionnaires. This questionnaire additionally inquired about HPs' views of the role of the training in changing their understandings and practices towards HCs. Post-training questionnaires were completed by 29 participants.

Pre-training and post-training questionnaires were perceived as lengthy and, at times, contained short or missing responses. The second group's responses were overall richer in information, as they were given more time to answer the pre-training questionnaires, and completed the post-training questionnaires in their own time. These participants submitted their responses via email (*n* = 12), resulting in missing data from two professional nurses. This also resulted in completion up to 2 weeks after the training, which for a few participants measured retained rather than immediate responsiveness.

Three to 4 months after the training, interviews were held with 10 purposively selected participants (four clinic managers, two senior professional nurses, one professional nurse, one environmental health practitioner and two health promotion officers). The criteria for selecting participants were based on their differences in sub-district and HC functioning. Besides contributing to the triangulation of earlier collected data, interviews further explored the role of the training on short-term HP responsiveness and their capacity to translate intentions to practices.

### Ethical Approval

The protocol was carried out in accordance with the recommendations of the Faculty of Health Sciences Human Research Ethics Committee of the University of Cape Town. The Faculty of Health Sciences Human Research Ethics Committee and the Health Department of the City of Cape Town approved this study (FHS HREC REF 2015/062 and ID no.: 10492, respectively). City of Cape Town sub-district managers permitted the recruitment of training participants for the evaluation. All subjects gave written informed consent in accordance with the Declaration of Helsinki. All training participants consented for the training to be observed (*n* = 34). Thirty-one participants agreed to complete the questionnaires and to be contacted for an interview. All 10 interviewees verbally consented to be contacted for follow up questions.

### Data Analysis

{NVivo 10} was used as a tool to manage all data. Questionnaire data was cleaned and anonymised in {Microsoft Excel} before being imported in{NVivo}. This also marked the start of the researcher's immersion in the data. Interviews were transcribed in {NVivo} and any text that could lead to the identification of the interviewee was removed. The first author was responsible for all data analysis and adopted a thematic approach as further detailed below.

Structural coding started with the mind mapping of questionnaire responses during preliminary analysis. These mind maps guided the inductive coding of topics and categories into an initial codebook. This codebook subsequently informed the initial codebook for the observations. Having mind-mapped the relationships between categories, and becoming familiar with the breadth of the investigated matter, the researcher examined the codes according to their ability to merge into categories and sub-codes. The data type-specific codebooks were refined and collapsed accordingly, which created small and simple codebooks with clear distinctions between the codes. Data was re-coded into meaningful units and the same was done for the remaining data, which slightly expanded the codebook again, after which the meaningful units were collapsed into themes. The first author discussed the emerging themes the second and third author of this paper. Similarly, as interviews were held 3–4 months after training these were coded sometime after the complete coding of the questionnaires and observations. This reflective pause allowed for emotional and intellectual distance, after which the sub-coded data were reorganised under more refined themes.

Ultimately, this resulted in a common codebook of three categorising codes that guided the answering of research questions, which were: understandings, practices, and intentions to change practice. Six main defining codes provided specifics to the categorising codes, which were; personal engagement with HC, HC roles and responsibilities, challenges and issues, stakeholders, role of training in changing responsiveness, and strategies to promote HC functioning. Thirdly, thematic codes and their descriptive sub-codes described the dimensions of the main and categorising codes. Moreover, data were grouped for analysis of pre- and post-training understandings, intentions and practices as well as classified for participant attributes such as reported HC functioning and professional position. A similar codebook was used for every data type, with differences in sub-codes, thematic codes and with, in some cases, additional main codes. Generally, themes and sub-codes, as well as their descriptions, maintained close similarity to the way in which participants phrased them.

Data triangulation and integrative analysis of themes arising from the different types of data guided the interpretations of the deeper meaning of codes, segments, and themes, respectively. After the entire analysis, thematic decisions were reflected upon as the first author's personal views of the themes could have changed. The diverse data revealing participants' changing reflections, understandings and intentions, as well as their ideas on the role of training, were compared to the first author's observations and rich, reflexive field notes.

A programme theory was used to frame the interpretive analysis of themes. This facilitated the assessment of the extent and nature to which HPs' post-training responsiveness outcomes, as defined by Lodenstein et al. (2017a), can contribute to the three interconnected principles of community participation, PHC and people-centred health systems as described in the introduction.

## Findings

### Current HC Presence and Engagement Practices

Of the 24 participants at clinic level, 17 indicated that the health facility is connected to a HC. All of these HCs consisted of community members and a facility manager, of which eight were said to be functioning well (by six clinic managers, one senior professional nurse, and one professional nurse). In two of these well-functioning HCs, environmental health practitioners were also included as members. A local government councillor was part of five HCs of which four were reported to be well-functioning. A little more than half of participants indicated that the facility regularly engages with the HC. Four clinic managers and two environmental health practitioners reported to never engage with the HC. All clinic managers had attended a HC meeting at least once before. Seven clinic managers, one senior professional nurse, two health promotion officers (a third of total participants) attend HC meetings each month. Ten participants (including all four professional nurses and two senior professional nurses) had never attended a HC meeting.

### HC Stakeholders

Following training, respondents' understandings of HC composition as defined by the NHA considerably increased (*n* = 29). Almost all respondents included community members (27 vs. 15, post-training and pre-training, respectively), local government councillors (27 vs. 10) and facility managers (25 vs. 7) in their description of composition. According to almost half of respondents, the HC composition, as stated by the NHA, should be complemented to include clinic workers other than the clinic manager. Some participants added that this would enhance communication and progress, this response was not related to the participant being positioned at the clinic. Environmental health practitioners were also viewed as important members by a third of respondents for their ability to address environmental problems that influence community health, such as illegal dumping.

When participants were explaining personal views on HC composition, a clinic manager expressed her concern:

“*My biggest challenge in the foreseeable future is to get a ward [local government] councillor, a proper ward councillor. Because this guy that's been the ward councillor for many years now for this area is very dedicated, is very well informed, has been a lawyer himself for many years so he's got a lot of background. And I think that's going to be very big shoes to fill.”*

Participants recurrently recommended that the HC engages with community stakeholders. For instance, schools, security guards, and social workers were seen to play a role in addressing major social problems, drug abuse and violence in the community. Additionally, non-profit and non-governmental organisations as well as churches in the area were perceived to promote awareness of the HC and avoid unnecessary duplication of health services. For the latter reason, a clinic manager without a HC expressed the intention to advise the sub-district's health promotion officer and programme coordinator, as well as non-governmental organisations to link with the HC.

### HC Roles and Responsibilities

To a greater extent than before the training, all participants made specific reference to the importance of HCs as liaison bodies between the wider community and the facility. It was underlined by nurses, clinic managers and a health promotion officer that HCs should inform the community of the challenges experienced by the facility and about the services that are offered to avoid unnecessary referral. In turn, the community was understood to benefit from the HC as a platform for advocacy. According to one of the clinic managers, the HC empowers the community to address and clarify their fears. It was also stated that HCs provide a true background of what needs to be done, and insight into how a community feels and thinks: “*They are able to reach where we can't”*. Several participants explained that HCs could identify the source of outbreaks (e.g., diarrhoea), mobilise the community to assist with campaigns, educate the community to prevent further spread and assist the health facility where needed. A clinic manager mentioned that the HC can facilitate a fast response of the services. Another clinic manager stated:

“*They are the people that are your ears and your eyes, but we tend to forget about them. […] They can make the decision or help us make the correct decision pertaining the community in which we serve.”*

Twenty-nine participants pointed out the health promotional and educational benefit that HCs could provide for the health facility. It was recurrently stated that HCs could assist with outreaches, inform the content of health talks at the clinic and help facilitate these. HC members could do home visits for the purposes of explaining home remedies, or for recalling patients. HC participation in promotional activities was repeatedly perceived to benefit clinic targets. One of the health promotion officers said*: “there are programmes that are not functional in the facility without the presence of the health committees, for instance the Health and Safety Committee”*.

Most participants understood that HCs can build and promote trust, facilitated by their interaction with both communities and facilities, their insight into the challenges at both levels as well as their ability to explain problems to the community and their closer relationship to them. Other reasons for HCs' role in trust building were transparency on what is being done at the facility and a sense of belonging for the community. A senior professional nurse said: “*This* [HCs] *is a great idea. The government has been spoon feeding the community for a very long time. It is now the time that the society takes the responsibility, or ownership of their health and this change* [implementing HCs] *would bring a tremendous improvement in our society because they do not feel left out.”*

HCs were also identified by half of participants as being able to assist with a smoother operation of the health care facility in easing tensions with the community, e.g., by helping with patient flow. Additionally, a couple of participants said the HC can set up a helpdesk at the facility, guide and fast track patients, as well as help management with the planning of health service transitioning, e.g., in the case where a clinic is transformed to a Community Health Centre. Moreover, HCs were commonly seen as beneficial to the facility as they can receive complaints, advise the facility on how to deal with these and lobby or help motivate for (the expansion of) resources. A clinic manager was convinced that services will improve and be used more as people are taking ownership of the health facility's decision-making.

Overall, the HCs' participation by means of these roles and responsibilities was commonly linked to contribute to the quality improvement, accessibility or responsiveness of service delivery to local needs.

### Perceived Role of Training

Most participants were surprised by the interactive nature of the training and reported a change in their perceptions as the training clarified HC roles and responsibilities. A clinic manager said: “*I feel the training was an eye-opener and empowering.”* As a result of the training, participants indicated they learned about the HC members and stakeholders, as well as the importance of all members' active involvement to be able to effectively implement their roles and responsibilities. Many participants specifically referred to not having known the local government councillor should be part of the HC as stated in the NHA. It also provided participants with insight into a HC's importance, other facilities' HP-HC working relationships, the need to appreciate HCs, their ability to address community problems through one platform and the accompanying opportunity for partnership in working towards a common goal. A senior professional nurse stated that “*the puzzle cannot be completed”* without HCs, as they play a key role in communicating between different community stakeholders. The majority of participants perceived HCs as an essential component in every community and facility, and confirmed a renewed insight into their importance, or perceived them as more valuable, post-training. The training was also said to facilitate a better understanding of what HCs should do and how to support HC functioning. According to a senior professional nurse, the training gave her a different perspective of the responsibilities of HCs, now making it easier to set boundaries. An environmental health practitioner no longer viewed the HC as a threat to HPs, as the training clarified that the facility managers' roles are not taken away from them. A clinic manager noted that the training teaches staff that HCs are not there to fight with HPs. Almost half of clinic managers found that their perceptions about HCs' roles and functions had not changed as a result of the training. Reasons being that HC already clearly outlined their roles or that they have always seen HCs as vital or valuable to the health facility's functioning. One of them added, “*… it is just that some of the health committee members did not have a clue of what their roles and responsibilities were when they were still functioning.”*

### Translating Understandings and Intentions to Practice

Clinic managers indicated that current barriers to their engagement with HCs are related to their own availability as well as their HC members' level of commitment. HPs' unavailability was, particularly among clinic managers and nurses, commonly explained by workload, having too many meetings already and HC meetings being held after hours. HC members' lack of commitment was repeatedly attributed to HC members having hidden agendas, being unavailable due to employment and not keeping to meeting times. A lack of funding was commonly identified as a challenge to retain HC volunteers, and for both HPs as well as community members to attend HC meetings. A clinic manager's view was that health committees should be funded regardless of whether they focus solely on HIV and tuberculosis care. Based on her observations, this explained different levels of HC functioning across sub-districts. Alternatives to monetary compensation were also considered, as a clinic manager illustrated:

“*…what came out for me also is how to motivate your community to take part. Not to just think of the money, but to think of something that is a stepping stone for them to maybe get a job. It is information that can go on their CVs at the end of the day. They gain experience, they gain knowledge, they meet new people. […] However, the negative of that is that people then sometimes expect to be placed in a position… Because of the high unemployment rate at the moment, people don't want to work for free. So there, we also need to then get people to become creative with how they can raise funds.”*

Other key challenges in working together with HCs were indicated to be a misunderstanding of and lack of mutual respect for one another's roles and responsibilities, leading to crossing boundaries and, consequently, mistrust. A clinic manager illustrated:

“*One of the negative things could be that HC members feel that they can work with clients and they have the authority to go through, to handle clients' folders, because they are the health committee.”* She advised: “*People [everyone at the clinic] should know their role and function, they [HCs] should be aware of their role and function, then we won't step on the other's toes.”*

Trust building was more generally perceived to be promoted by training and clear guidelines on roles and functions, providing the HC with an opportunity to present on their roles and responsibilities, maintaining honesty as well as establishing a common vision at the start. Furthermore, HPs identified HCs' attitudes and judgment as challenging to their relationships. As expressed by a clinic manager: “*I think if both of us [HPs and community members] have a positive attitude towards one another, we can move mountains.”* Moreover, power differences were recurrently understood to be resolved by understanding and setting clear cut roles and responsibilities, ensuring transparency and sharing power equally. As formulated by a clinic manager: “*Do not* run*, do not make me your subordinate, but make me somebody that you work* with*, then I think we could function.”*

Five participants stated that they would not engage differently as they were already dealing with community related issues, the HC was already functioning well, or they had established a relationship in which there was an awareness of boundaries. Other reasons for not being completely convinced of different personal engagement were the HCs' lack of visibility in the participants' current position and the need for time off work to engage with them. In contrast, a programme officer indicated that she would assist the facility managers in the HC role, even though she was not working with HCs herself. Nurses said they would consult the HC about ways to improve health talks, to provide more guidelines regarding HC functions, and to involve them in decision-making pertaining to the community. Clinic managers intended to actively participate in meetings more regularly, request help in various work areas, and to invite the local government councillor to assist in establishing a new committee. Another clinic manager said that the training, because it provided self-development and stimulated an awareness of what is happening at other facilities, made her willing to improve her relationship with other HC stakeholders. Other participants intend to encourage the HC to run a helpdesk and facilitate active co-operation between all HC members and HPs.

Two clinic managers contacted the local government councillor regarding the HC as a result of the training. In one instance, it had not yet been possible to reach the councillor, and in the other, the councillor would connect the clinic manager to an active community member who could become involved. Aside from these two cases, most intentions to change practices remained intentions for the duration of this evaluation, with some participants feeling constrained by their superiors. A clinic manager pointed out that her manager questioned her training attendance because of her workload. Some participants therefore perceived it relevant to train other health care providers, such as the sub-district managers and the second in charge. An environmental health practitioner said: “*At the beginning I was told not to make myself clever. […]I don't think I can take initiative on this, because it's not part of my work.”*

## Discussion

This section discusses the above reported post-training responsiveness of HPs to HCs as community-based governance structures. Further, we shed light on the potential contribution of HPs' enhanced responsiveness to the people-centeredness of health systems. Moreover, study and training limitations as well as recommendations are described.

The evaluated HP training cross-cuts the six key mechanisms to HP responsiveness identified in Lodenstein et al. (2017a) realist review of social accountability initiatives in LMICs. In their proposed programme theory, the authors suggest that HP responsiveness outcomes are influenced by (i) HPs' perceptions on the legitimacy of the social accountability initiative, (ii) their feelings of support, safety, appreciation, and (iii) of moral responsibilities and obligations, (iv) their fear for public or professional reprisal, (v) their self-identification with the initiative's claims or ideals and perceived self-capacity to act, and (vi) their perceptions on health care users. The authors categorise HP responsiveness outcomes as “receptivity,” “relations,” and “responsiveness.” In Table 3, the earlier described themes and findings on HP reported post-training understandings, intentions and practices towards HC functioning are organised by these HP responsiveness outcomes (Lodenstein et al., 2017a). The contextual factors of potential influence as identified by HPs and, to a lesser extent, in the discussion below are also summarised in this table.

**Table 3:** Summary of themes and findings on HP post-training responsiveness to HCs and contextual influences.

**HP reported post-training responsiveness**	**Understandings of HC roles and functions**	**Intentions to engage with HCs**	**Practices towards HC functioning**	**Identified contextual influences**
**Receptivity**	- Local government councillor as part of the HC - Importance of engagement with other community stakeholders - Mutual importance and benefit of HC as a liaison body for HPs and community - HC roles and responsibilities - How to support the HC	- Invite local government councillor to assist with new HC establishment - Assist clinic manager with HC roles	- Contacted local government councillor	- Mutual respect - Understandings of roles and responsibilities - Crossing of boundaries - Trust - HC attitude and judgment
**Health service responsiveness**	- Avoid duplication of health services and inform patients on services offered - Identify outbreak sources —prevent spread - Fast response of services - Community mobilisation for campaigns - Assist with health promotion and patient recall - Management of patient flow - Help with: decision-making regarding community, planning health service transitions, complaint management, resource motivation	- Consult HC to improve health talks - Involve HC in decision-making regarding community - Encourage HC to run helpdesk	*Not evaluated*	- Broader community awareness of HC presence and roles - Representativeness of members^*^
**Relations**	- Importance of all members' active involvement - Have a common vision from the beginning and partnership working towards it - Clear cut roles and responsibilities - Need for appreciation of HCs - Trust-building, transparency, honesty - Community ownership - Easing tensions - Community and facility awareness of HC presence and roles - Role of training - Sharing power equally	- Regular, active meeting participation - Facilitate active cooperation between HC members and HPs - Provide more guidelines regarding HC roles	*Not evaluated*	- HC member commitment and availability - HC member retention - Repeated training of HCs^*^ - Individual agendas - Governmental priorities^*^ - Hierarchy - Current position held - Guidelines - Policy and legal framework^*^ - Workload - Transport money - Fund raising initiatives - System funding priorities - Clinic manager availability (workload, after hour-HC meetings, other meetings)

* *Identified in the discussion*.

### Receptivity

“Receptivity” is the collection of attitudes, awareness and acceptance around the social accountability initiative. Following training, HCs were increasingly perceived as beneficial to the facility, besides their benefit to the community. HPs increased responsiveness towards HCs' roles and responsibilities consisted of improved understandings of what these roles and responsibilities are and how to support them. Furthermore, HPs increasingly welcomed the active involvement of the local government councillor and the environmental health practitioner as HC members, as well as engagement with other community stakeholders. This receptivity can form a foundation for enhancing relations and health services responsiveness outcomes, thereby facilitating the availability of health services, mobilisation or reallocation of resources and targeting the environmental and socio-economic determinants of health. This would contribute toward the holistic approach of people-centred health care and systems and the right of everyone to a complete state of social, mental and physical well-being (Backman et al., 2008; WHO, 2015). However, HP receptivity to HCs is subject to HP identified contextual influences such as mutual respect, trust and (feeling subjected to) HC community members' judgment.

### Health Service Responsiveness

To avoid confusion with our own definition of HP responsiveness, we refer to responsiveness as the outcome of HP responsiveness as “health service responsiveness.” It is defined by the concrete actions towards improving service provision in line with citizen concerns (Lodenstein et al., 2017a). HPs contribute to people-centred, needs-responsive healthcare when they provide an enabling environment in which the community is truly represented and participating, can take control of their own health and provided a platform to engage with the health system as a whole (WHO, 2015). HPs showed enhanced understandings and intentions towards the participation and representation of the community in the setting of goals, decision making and problem solving regarding their identified needs, concerns, and expectations. The extent to which the needs-responsiveness of local health services can be improved will be influenced by the level of awareness of HC presence and roles amongst the broader community as well as the extent to which HC members are representative of the community that the facility serves. The exact extent to which HCs would be promoted to actively participate in the monitoring, strategizing, and planning of service delivery, and the weight of their participation in the decision-making remains unclear and is dependent of HP-HC relations.

### Relations

The “relations” outcome is described by the changes in interactions and accountability between communities and HPs. This outcome appears to be the most dynamic in nature. Similarly to the other HP responsiveness outcomes, the relations outcome influences and is influenced by the other two outcomes. Following training, HPs expressed understandings and intentions towards the active involvement of all HC members, their own regular active participation in meetings, and strategies for the building and strengthening of relationships. Such understandings of strategies promotive of HCs' role in building trust included the setting of clear roles and responsibilities, appreciating HCs and working towards a common vision in equal partnership. These HP understandings and intentions can facilitate an enabling environment and opportunities for community members to meaningfully participate in the strengthening of governance and accountability within the health system. Hence, HP enhanced responsiveness following training can play a role in decreasing the “participatory deficit” in the way health services are planned and delivered, contributing to the people-centeredness of service delivery (WHO, 2015). The HP-community relations shape the environment in which HP receptivity to HCs can be influenced and determine the extent to which the community participates, subsequently influencing health care responsiveness outcomes. Moreover, HP-community relations are in itself influenced by e.g., the feasibility of HC meeting times for HPs and priorities of a variety of actors across the system, including HC community members and supervisors.

### Potential Role of Contextual Influences

When provincial priorities in the Eastern Cape changed, health promotion managers' and health advisors' role to develop, establish and support HCs were only maintained in a few cases after reviving these roles (Boulle et al., 2008). As identified in this evaluation, unresponsive superiors can impede HPs' ability to engage with the HC. In Kenya, sustainability and replication of HC success required more than once-off training of both community members and health staff, continuous follow-up, as well as commitment from district level authorities (Sohani, 2005). A legal framework recognising and specifying HCs' full capacity as well as some funding to facilitate transport costs for meetings may also be required to build sustainable working relationships between HPs and HCs. However, the opposite can be true. In Guinea, a HC's functioning was attributed to the intrinsic motivation of its members and a HC in the DRC developed their own operational guidelines and received voluntary community contributions (Lodenstein et al., 2017b). This evaluation has shed light on the current presence of HPs who are already responsive to HCs, have promoted HC functioning or have good relationships with HCs irrespective of political agendas. Furthermore, evidence from Sub-Sahara Africa and Bangladesh (Knox, 2009; Tembo, 2013) shows that although policy and legal frameworks are important in formalising HC power and mandate, their utility can be limited in the absence of a basic level of trust. This suggests that HPs increased responsiveness to community engagement can positively influence opportunities for the community to participate and facilitate HC functioning despite crosscutting issues and contextual factors affecting their functioning.

Even though there are presumed plans for a national roll out of HC training through the training of trainers, provincial and national policies appear to only move further away from meaningful community participation in the delivery of PHC (Province of Western Cape, 2016; The Republic of South Africa, 2018). The current nature of implementation of health reforms, the lack of defined allocated power to HCs and the limitations to HC member selection points at the technocratic nature of participation in the South African health system. If South Africa is to achieve “universal health coverage” with its National Health Insurance Bill approaching finalisation, it is important to underline and advocate for the role HP-HC relationships and HP capacity building can play in the decision-making, planning, and implementation as well as strengthening of needs-responsive and people-centred PHC. However, we acknowledge that despite HP training, HCs will be subject to other contextual influences while navigating the system. Hence, HP training should be part of a concerted effort to improve HC functioning.

### Role of Training

In short, HP training that adopts a rights-based, interactive approach, and cuts across the six mechanisms for change of HP responsiveness to social accountability initiatives, can promote HP responsiveness outcomes. This study contributes to the conceptualisation of the programme theory proposed by Lodenstein et al. (2017a). The training facilitated HPs', receptivity to HCs and it also provided HPs with the understandings and skills to develop appropriate intentions and, in some cases, implement practices towards building HC relations and health care responsiveness.

This evaluation confirmed HPs' key role in the functioning of HCs and provides momentum for the wider investigation of the role of such rights-based, interactive training in promoting social accountability initiatives. In light of our findings, we recommend that HP long-term responsiveness to HCs following training is also evaluated and the training is tested in other contexts. Considering the ever-changing relationship dynamics and contextual influences, both HPs and community members could benefit from iterated interactive training over time. For this purpose, the value and feasibility of jointly organised follow up-sessions is worth exploring further.

### Limitations

This study may have been subjected to an inclusion bias. HPs' ability to attend the training could have been influenced by the priorities and workload of their respective clinic or sub-district at the date of training. Besides, only HPs linked to “City of Cape Town clinics” were included in this study. City of Cape Town clinics differ from other clinics in the Cape Metropole as they are managed by the municipality instead of the provincial government. Historically, these clinics were more health promotional, preventative and community-oriented. The provincially managed health facilities originally delivered curative services only. Some HPs could therefore have already been more responsive to the concept of community participation. Another current difference is that City of Cape Town clinics are managed and run by nurses only.

The changes to training implementation had consequences on the comprehensiveness of the evaluation and may have impacted rigour. Despite willingness from the participants, the lack of opportunity to follow-up on the first training session could illustrate that competing priorities are a challenge in committing to the full, intended training programme. It would therefore be worth exploring the socio-political and economic influences on cross-health system level stakeholders' responsiveness to community participation, HCs and HP training on community participation through HCs.

The study was constrained by time. Ideally, a longer evaluation period could have allowed for time to translate, pilot and validate the questionnaires. The absence of the translation of our methods into most participants' first language limited the cultural acceptability of our methods, potentially losing out on local meaning and cultural connotations. Nonetheless, the questionnaire responses were consistent with the training observations and there was room for the exchange of cross-cultural and -lingual understandings during training sessions and semi-structured interviews. A longer study period could have facilitated a push for the rescheduling of follow-up sessions. Participants raised issues for discussion in these sessions which could have further developed HPs' practical skills to establish a common vision, host a HC meeting and manage conflict. This can be a limitation to the implementation of participants intended strategies. It would also be recommended to further investigate the role of training on the long term responsiveness of HPs in building working relationships with HCs and on HC functioning. For instance, a documentary review of the HC meeting minutes was initially proposed in order to assess HPs' pre and post-training interactions with HCs.

## Conclusion

Interactive, rights-based training of HPs on community engagement can enhance HP responsiveness to HCs. As a result of this training, HPs were receptive of HCs as community-based accountability structures. Furthermore, they demonstrated understandings of and intentions towards the strengthening of HP-HC relationships and the promotion of HC roles and responsibilities in the delivery of PHC. HPs' responsiveness following training can facilitate HC potential to improve the needs-responsiveness of PHC and the people-centeredness of health systems. Considering the contextual influences that HP responsiveness can be subjected to, this training should be tested and evaluated further.

## Author Contributions

GZ designed the study, collected, and analysed the data. MS and HH provided feedback and input throughout these stages. GZ wrote the manuscript on which MS, HH, and LL provided feedback.

### Conflict of Interest Statement

The authors declare that the research was conducted in the absence of any commercial or financial relationships that could be construed as a potential conflict of interest.

## Abbreviations

HC: Health Committee
HP: Health Care Provider
LMICs: Low- and Middle-Income Countries
PHC: Primary Health Care
NHA: National Health Act
WHO: World Health Organisation.
